# The Comparison of Insulin Resistance Between Normal and Early Menopause Women Younger than Fifty Years Old by Machine Learning Methods

**DOI:** 10.3390/diagnostics15162074

**Published:** 2025-08-19

**Authors:** Chun-Kai Wang, Dee Pei, Ta-Wei Chu, Kai-Jo Chiang

**Affiliations:** 1 Department of Obstetrics and Gynecology, Zuoying Armed Forces General Hospital, Kaohsiung 813, Taiwan; oneway1982@yahoo.com.tw; 2 School of Medicine, National Defense Medical University, Taipei 114, Taiwan; 3 Division of Endocrinology and Metabolism, Department of Internal Medicine, Fu Jen Catholic University Hospital, School of Medicine, College of Medicine, Fu Jen Catholic University, New Taipei 243, Taiwan; peidee@gmail.com; 4 Department of Obstetrics and Gynecology, Tri-Service General Hospital, National Defense Medical University, Taipei 114, Taiwan; taweichu@gmail.com; 5 MJ Health Research Foundation, Taipei 114, Taiwan; 6 School of Nursing, National Defense Medical University, Taipei 114, Taiwan; 7 Department of Nursing, Tri-Service General Hospital, Taipei 114, Taiwan

**Keywords:** machine learning, early menopause, Taiwanese women, insulin resistance, multiple linear regression, homeostasis assessment of insulin resistance

## Abstract

**Background**: The prevalence of type 2 diabetes (T2D) is on the rise, and insulin resistance (IR) is one of the key risk factors for developing T2D. This paper seeks to identify risk factors for IR in women with normal menstrual cycles (NM) and early menopausal women (EM). **Methods**: EM women between 30 and 50 years old were compared with an NM control group. Four machine learning (ML) methods were trained using comprehensive physiological and lifestyle data to estimate a homeostasis model for insulin resistance (HOMA-IR dependent variable). Traditional multiple linear regression (MLR) was used as a benchmark for comparison. **Results**: A total of 948 participants were enrolled (NM: 410, EM: 538). On average, ML outperformed MLR, identifying the six key risk factors in the EM group (from most to least important) as waist–hip ratio (WHR), triglyceride (TG), glutamic-pyruvic transaminase (GPT), glutamic oxaloacetic transaminase (GOT), HDL-Cholesterol (HDL-C), and lactic dehydrogenase (LDH). Rankings differed in the NM group, with WHR identified as the leading risk factor, followed by C-reactive protein (CRP), HDL-C, total bilirubin (TBIL), diastolic blood pressure (DBP), and white blood cell count (WBC). **Conclusions**: Using ML, we found that WHR and HDL-C are the common denominators in both EM and NM women, with additional correlations with TG, liver enzymes and LDH for EM women. These results clearly indicate the importance of estrogen protection, suppressing less important factors (TG, liver enzyme, and LDH), and only the stronger inflammatory markers become important (CRP, TBIL, and WBC). Once estrogen’s protection disappears, the suppression of CRP, TBIL, and WBC would become weaker. Since these 3 features are significantly correlated with body weight, for women under 50, reducing body weight is the most important factor in preventing hyperglycemia.

## 1. Introduction

Global prevalence of type 2 diabetes (T2D) has increased significantly in recent decades, with current World Health Organization estimates of 800 million individuals, or 14% of the world adult population [1]. The two main pathophysiologies for T2D are increased insulin resistance (IR) and decreased insulin secretion [2].

The average age of menopause in Taiwan ranges between 49.5 and 50.2 years old [3,4]. However, many women undergo early menopause (EM) before the age of 50, with increased risk of T2D. EM can result from chromosome abnormalities, autoimmune disorders, chemotherapy, smoking, and low BMI [5,6,7]. Early onset of menopause, especially before the age of 45, has been linked to metabolic abnormalities such as heightened insulin resistance (IR), unfavorable lipid profiles, and an increased risk of cardiovascular disease. Several studies have documented a rise in IR following menopause due to changes in estrogen levels, body composition, and inflammatory markers [8,9].

Studies such as the Kinmen Study have demonstrated that menopause independently contributes to the development of metabolic syndrome and IR, even in nondiabetic women [10]. Furthermore, randomized trials like PEPI and HERS have shown estrogen replacement therapy improves fasting insulin levels and homeostasis assessment model-IR scores, underlining the hormonal influence on metabolic regulation [11].

EM has been associated with a higher risk of developing T2D and IR. A meta-analysis published in Nutrition & Metabolism found that EM women had a 24% higher risk of developing T2D, likely due to reduced lifetime exposure to estrogen and associated metabolic dysregulation [12,13].

While traditional statistical methods have been widely used to evaluate IR, machine learning (ML) has emerged as a powerful tool for identifying complex, nonlinear patterns in biomedical data. Xing et al. (2024) applied ML algorithms to NHANES data to classify IR in middle-aged women, achieving high accuracy and identifying body mass index (BMI), triglycerides, and HbA1c as important predictors [14]. More recent studies have used deep learning or ensemble models (e.g., CatBoost and XGBoost) and even wearable sensor data to predict IR in real time [15,16].

To the best of our knowledge, no previous study has combined stratification by menopausal timing (normal vs. early) with ML-based modeling of IR in women under 50. Our study aims to fill this gap by using machine learning to compare insulin resistance patterns between these two groups, potentially offering new options for early identification and intervention strategies for at-risk women.

The present study:

1. Compares the performance between traditional multiple linear regression (MLR) and ML.
2. Uses ML to identify risk factors for IR between women with normal menstrual cycles (NM) and EM.
3. Uses Shapley addictive explanation (SHAP) to understand individual-level prediction errors and identify areas of might underperformance.

## 2. Materials and Methods

### 2.1. Participants and Study Design

The participant recruitment process and study design closely mirrored those of our previous research [17]. Data were obtained from the Taiwan MJ cohort, an ongoing prospective cohort derived from health examinations conducted at the MJ Health Screening Centers across Taiwan. These examinations encompass over 1000 key biological indicators, including anthropometric measurements, blood analyses, and imaging assessments. Each participant completed a self-administered questionnaire capturing personal and family medical history, current health conditions, lifestyle behaviors, physical activity, sleep patterns, and dietary habits. Participant data were provided by the MJ Clinic. General consent for participation in future anonymous research was obtained at the time of health screening. The resulting database is curated and maintained by the MJ Health Research Foundation, an affiliate of the MJ Clinic, which authorized data access for this study (Authorization Code: MJHRF2024018A). The interpretations and conclusions presented herein are solely those of the authors and do not reflect the views of the Foundation. As this study involved secondary analysis of existing data without new sample collection, additional informed consent was not required. For detailed data collection procedures, please refer to the MJ Health Research Foundation’s annual technical report [18]. The study protocol was reviewed and approved by the Institutional Review Board of Kaohsiung Armed Forces General Hospital (IRB No.: KAFGHIRB113-002). Given the nature of secondary data analysis, only an expedited review process was necessary.

**Figure 1:**
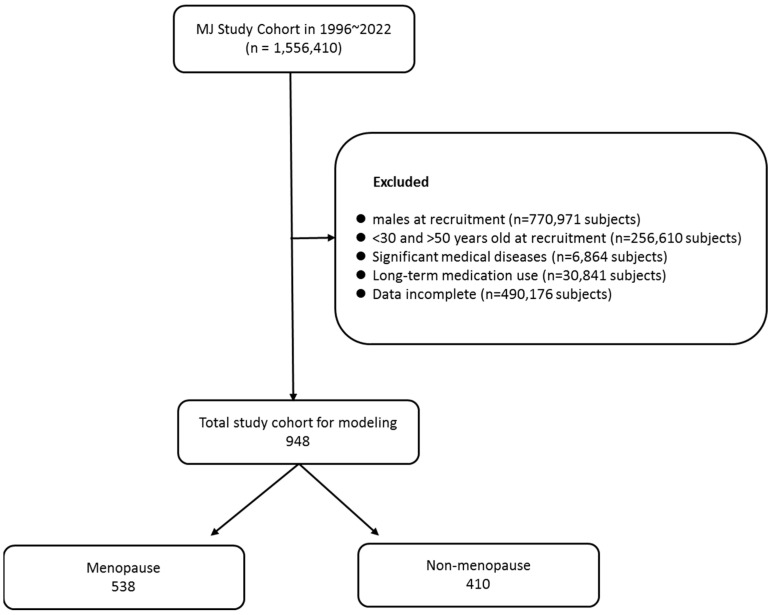
Study participant selection.

The initial sample pool included 1,556,410 subjects. After excluding subjects who did not fit our criteria, a final sample of 948 women between 30 and 50 years old were enrolled (538 EM, 410 NM) (Figure 1). The inclusion criteria were:

1. No history of significant medical diseases such as stroke, myocardial infarction, or heart failure;
2. No diagnosis of diabetes;
3. No medication for metabolic syndrome.

### 2.2. Anthropometry and Biochemistry Measurements

For further details regarding measurement protocols, please refer to our previous publication [19]. On the day of examination, a senior nurse recorded each participant’s personal history, including education level and consumption habits related to tobacco, alcohol, and betel nut. Body weight (kg) was measured using an electronic scale, while systolic and diastolic blood pressures (SBP and DBP) were assessed with an automatic sphygmomanometer.

Fasting blood samples were collected after a 10 h overnight fast. Plasma was separated within one hour of collection and stored at −30 °C for lipid analysis. Total cholesterol and triglyceride (TG) levels were determined using the dry multilayer analytical slide method with the Fuji Dri-Chem 3000 analyzer (Fuji Photo Film, Tokyo, Japan). Serum concentrations of high-density lipoprotein cholesterol (HDL-C) and low-density lipoprotein cholesterol (LDL-C) were measured using an enzymatic cholesterol assay following dextran sulfate precipitation.

#### Traditional Statistical Analysis

Data are presented as the mean ± standard deviation. Student’s *t*-test was employed to compare the NM and EM groups, while analysis of variance (ANOVA) was used to assess ordinal variables such as education and income level. Pearson’s correlation coefficient was calculated to examine the relationships between continuous variables and insulin resistance (IR), as assessed by the homeostasis model assessment of insulin resistance (HOMA-IR). Multiple linear regression (MLR) was used as a baseline model for comparison with machine learning (ML) methods. All statistical analyses were two tailed, with a significance threshold set at *p* < 0.05. Analyses were conducted using SPSS version 10.0 for Windows (SPSS Inc., Chicago, IL, USA).

### 2.3. Study Dataset

Table 1 provides the mean ± standard deviation for 33 (independent) clinical variables. Drinking area was defined as the multiple of total alcohol consumption duration, consumption frequency and alcohol content percentage. Similarly, smoking area was defined as the multiple of the duration and frequency of smoking and number of cigarettes consumed. Sport area was the multiple of duration, frequency, and type of exercise. Sleep time was an ordinal variable. IR was measured using the homeostasis model assessment as a surrogate (HOMA-IR) which is the dependent variable. Fasting plasma glucose and insulin levels were not included in the statistical analysis since HOMA-IR was calculated from these two parameters (dependent variable).

Equation used to calculate HOMA-IR

$$HOMA-IR=\frac{FPI(\mu U/mL)\times FPG(mg/dL)}{405}$$

### 2.4. Proposed Machine Learning Scheme

Four machine learning (ML) algorithms—random forest (RF), stochastic gradient boosting (SGB), eXtreme Gradient Boosting (XGBoost), and Elastic Net (EN)—were employed to develop predictive models for HOMA-IR. These algorithms have been extensively applied in healthcare research and are advantageous in that they do not require assumptions about the underlying data distribution [20,21,22,23,24,25,26,27,28]. Further methodological details are available in our previous publication [29].

RF is an ensemble learning method based on decision trees that utilizes bootstrap resampling and bagging techniques [30]. It constructs numerous unpruned classification and regression trees (CARTs), with splits determined by minimizing Gini impurity. These trees are aggregated to form a “forest”, and the final prediction is derived by averaging the outputs (for regression) or through majority voting (for classification), thereby enhancing model robustness and reducing overfitting [31].

SGB is a tree-based gradient boosting approach that integrates bagging and boosting to minimize loss and address the overfitting limitations of traditional decision trees [32,33,34]. It generates a series of weak learners (trees) in a sequential manner, where each tree is trained to correct the residual errors of its predecessor. This iterative refinement continues until a specified stopping criterion—such as the maximum number of iterations or model convergence—is met. The final output is the aggregated result of all the trees.

XGBoost is an optimized implementation of gradient boosting, offering significant improvements over traditional SGB in terms of speed and predictive accuracy [34]. It constructs ensembles of weak learners using a gradient boosting framework, incorporating a second-order Taylor expansion to approximate the objective function. XGBoost supports custom differentiable loss functions and includes a regularization term to control model complexity, thereby improving generalizability and preventing overfitting [35].

EN is a linear model that incorporates both L1 (Lasso) and L2 (Ridge) regularization penalties. It effectively combines variable selection and regularization by enabling sparse model coefficients (as in Lasso) while preserving the stability of Ridge regression. EN is particularly useful when predictors are highly correlated, as it encourages the inclusion of grouped variables rather than arbitrarily selecting one, which is a common limitation of Lasso alone [36].

All continuous variables were standardized using z-score transformation (mean = 0, standard deviation = 1) prior to training the EN and MLR models to ensure comparability of coefficients and optimize convergence. For tree-based algorithms (RF, SGB, and XGBoost), no standardization was applied, as these models are scale-invariant and do not require feature normalization.

**Figure 2:**
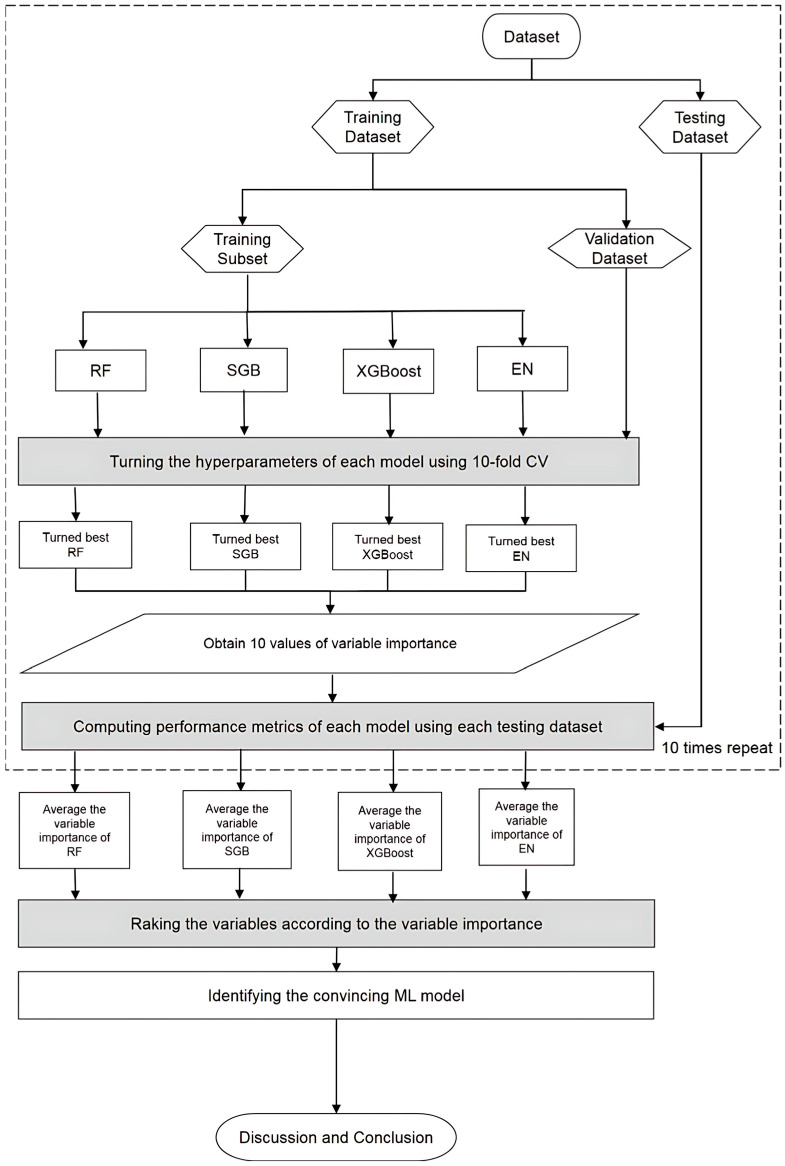
Flowchart of the proposed scheme.

Figure 2 illustrates the flowchart of the proposed predictive modeling and variable importance identification framework that integrates four ML algorithms. The process began with the collection of patient data, which were then preprocessed and structured into a dataset following the proposed methodology. The dataset was randomly split into two subsets: 80% for training and 20% for testing. During the training phase, each ML algorithm was optimized through hyperparameter tuning to enhance model performance. This was achieved using a 10-fold cross-validation (CV) approach, where the training set was further divided into training and validation subsets. A comprehensive grid search was performed across all feasible combinations of hyperparameters. For each algorithm, the model yielding the lowest root mean square error (RMSE) on the validation subset was selected as the optimal model. As a result, well-tuned versions of the RF, SGB, XGBoost, and EN models were obtained. These models were then used to evaluate predictive performance on the testing dataset, and variable importance rankings were extracted to identify key predictors associated with the outcome of interest.

The testing dataset was utilized to evaluate the predictive performance of the RF, SGB, XGBoost, and EN models. Given that the target variable in this study is continuous, model performance was assessed using several regression metrics, including symmetric mean absolute percentage error (SMAPE), relative absolute error (RAE), root relative squared error (RRSE), and root mean squared error (RMSE). A summary of these evaluation results is presented in Table 2.

To ensure a robust comparison, the training and testing processes mentioned above were repeated randomly for 10 iterations. The averaged metrics of the RF, SGB, XGBoost and EN models were then used to compare model performance against the benchmark MLR model using the same training and testing datasets as the ML methods. An ML model with an average metric lower than that of MLR was considered a convincing model.

As all ML methods employed in this study are capable of ranking predictor variables by importance, each variable was assigned a priority score ranging from 1 to 30 within each model, in descending order of importance. However, due to inherent differences in algorithmic structure and feature selection mechanisms, the variable importance rankings may vary across models. To improve the robustness and consistency of the final rankings, we aggregated the variable importance results from the most reliable ML models.

In the final phase of the proposed framework, we synthesized and interpreted the key findings derived from these selected ML models. This integrative approach facilitated the identification of variables that play a substantial role in predicting HOMA-IR.

All ML analyses were conducted using R software version 4.0.5 and RStudio version 1.1.453, with the necessary packages installed [37,38]. Specifically, RF, SGB, and XGBoost were implemented using the “randomForest” package (v4.6-14) [39], “gbm” package (v2.1.8) [40], “rpart” package (v4.1-15) [41], and “xgboost” package (v1.5.0.2) [42], respectively. The “caret” package (v6.0–90) [43] was utilized to perform hyperparameter tuning and model optimization for RF, SGB, XGBoost, and EN. The multiple linear regression (MLR) model was implemented using the base “stats” package in R (v4.0.5), applying default settings. SHAP was conducted using the following Python packages 3.13.7; SHAP, the core package for computing and visualizing SHAP values, provides interpretability for model predictions and feature importance. Pandas, a powerful library for data manipulation and preprocessing, was used to manage datasets, clean data, and prepare inputs for SHAP analysis. NumPy, a fundamental package for numerical computations, supported array operations and numerical calculations required by SHAP. Matplotlib 3.10.3, a plotting library for creating static, interactive, and animated visualizations, was used to generate SHAP plots, including summary plots, bar plots, and waterfall plots to highlight contributions to specific predictions.

## 3. Results

**Figure 3:**
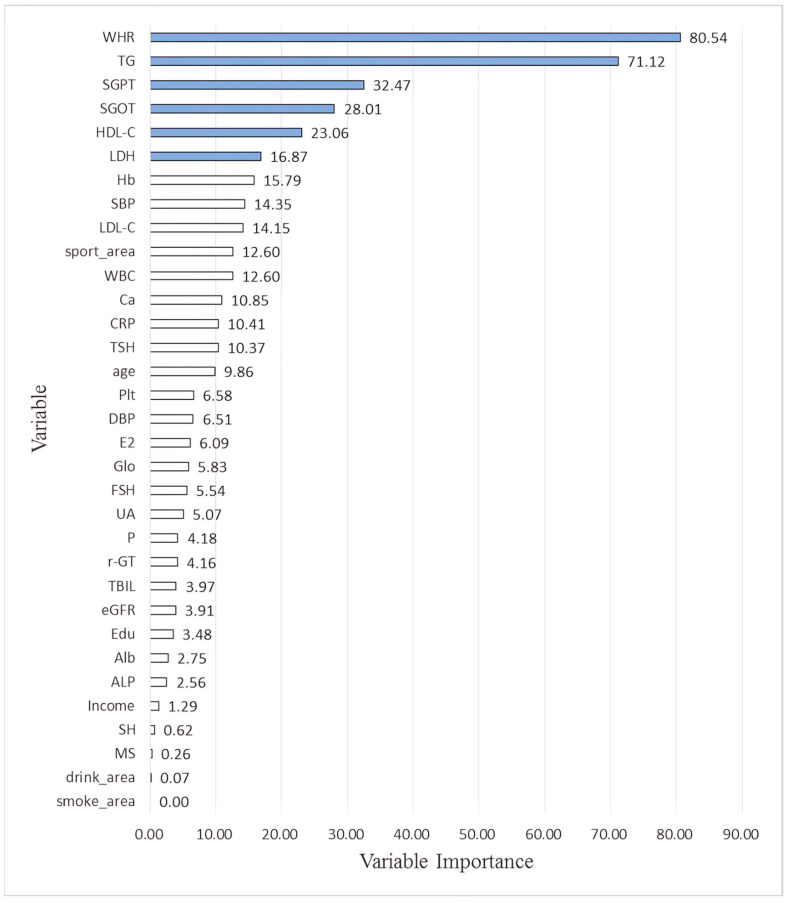
Graphic representation of the percentage of importance selected by machine learning methods related to HOMA-IR (Normal Menopause). The blue bars are the most six features. Note: The full names of the variables abbreviations on the *Y*-axis of the chart are as follows: WHR: Waist–hip ratio; TG: Triglyceride; SGPT: Serum glutamic pyruvic transaminase; SGOT: Serum glutamic oxaloacetic transaminase; HDL-C: High-density lipoprotein cholesterol; LDH: Lactate dehydrogenase; Hb: Hemoglobin; SBP: Systolic blood pressure; LDL-C: Low-density lipoprotein cholesterol; WBC: Leukocyte; Ca: Plasma calcium concentration; CRP: C reactive protein; TSH: Thyroid-stimulating hormone; Plt: Platelets; DBP: Diastolic blood pressure; E2: Estradiol; Glo: Globulin; FSH: Follicle-stimulating hormone; UA: Uric acid; P: Plasma phosphate concentration; r-GT: γ-Glutamyl transpeptidase; TBIL: Total bilirubin; eGFR: estimated Glomerular filtration rate; Edu: Education level; Alb: Albumin; ALP: Alkaline phosphatase; SH: Sleeping hours; MS: Marital status.

**Figure 4:**
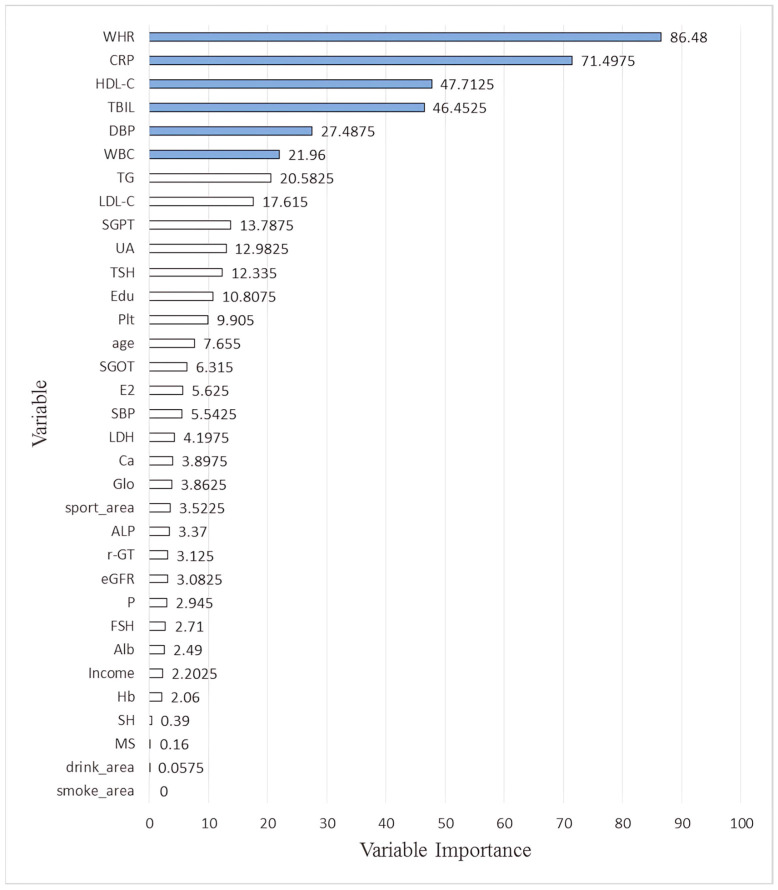
Graphic representation of the percentage of importance which were selected by machine learning methods related to HOMA-IR (non-menopause). The blue bars are the most six features. Note: The full names of the variables abbreviations on the *Y*-axis of the chart are as follows: WHR: Waist–hip ratio; CRP: C reactive protein; HDL-C: High-density lipoprotein cholesterol; TBIL: Total bilirubin; DBP: Diastolic blood pressure; WBC: Leukocyte; TG: Triglyceride; LDL-C: Low-density lipoprotein cholesterol; SGPT: Serum glutamic pyruvic transaminase; UA: Uric acid; TSH: Thyroid-stimulating hormone; Edu: Education level; Plt: Platelets; SGOT: Serum glutamic oxaloacetic transaminase; E2: Estradiol; SBP: Systolic blood pressure; LDH: Lactate dehydrogenase; Ca: Plasma calcium concentration; Glo: Globulin; ALP: Alkaline phosphatase; r-GT: γ-Glutamyl transpeptidase; eGFR: estimated Glomerular filtration rate; P: Plasma phosphate concentration; FSH: Follicle-stimulating hormone; Hb: Alb: Albumin; Hb: Hemoglobin; SH: Sleeping hours; MS: Marital status.

A total of 948 participants were enrolled (NM: 410, EM: 538). Their demographic, biological, and lifestyle data are summarized in Table 3 (mean ± standard deviation). The NM group had lower values for WBC, platelet, estradiol and higher hemoglobin, total bilirubin, albumin, alkaline phosphatase, γ-glutamyl transpeptidase, lactate dehydrogenase, uric acid, TG, LDL-C, C, P phosphate, and follicular-stimulating hormone. The two groups showed no differences in terms of marital status, income, education level, sleeping hours, or sport, drinking and smoking area. For some features, *n* does not sum up to the total partipicants number (948) due to missing data. Table 4 shows the simple correlation results, with HOMA-IR positively correlated with WHR, SBP, DBP, WBC, Hb, Plt, FPG, Alb, Glo, ALP, SGOT, SGPT, γ-GT, LDH, UA, TG, LDL-C, Ca, CRP and negatively correlated with TBIL, HDL-C, P, FSH and sport area. Table 2 presents the equations for performance metrics. Table 5 presents the average performance of MLR and the other four ML methods. All estimation errors were smaller for the ML methods, indicating usperior performance. Finally, the performance percentage from the four ML and their averages are shown in Table 6 and Table 7 for the NM and EM groups. Since they are not sorted according to their number, Figure 3 and Figure 4 provide a grahic representation after sorting according to their dimensions. For EM, the most important six factors were WHR, TG, GPT, GOT, HDL-C and LDH, while those for the NM group were WHR, followed by CRP, HDL-C, TBIL, DBP and WBC.

**Figure 5:**
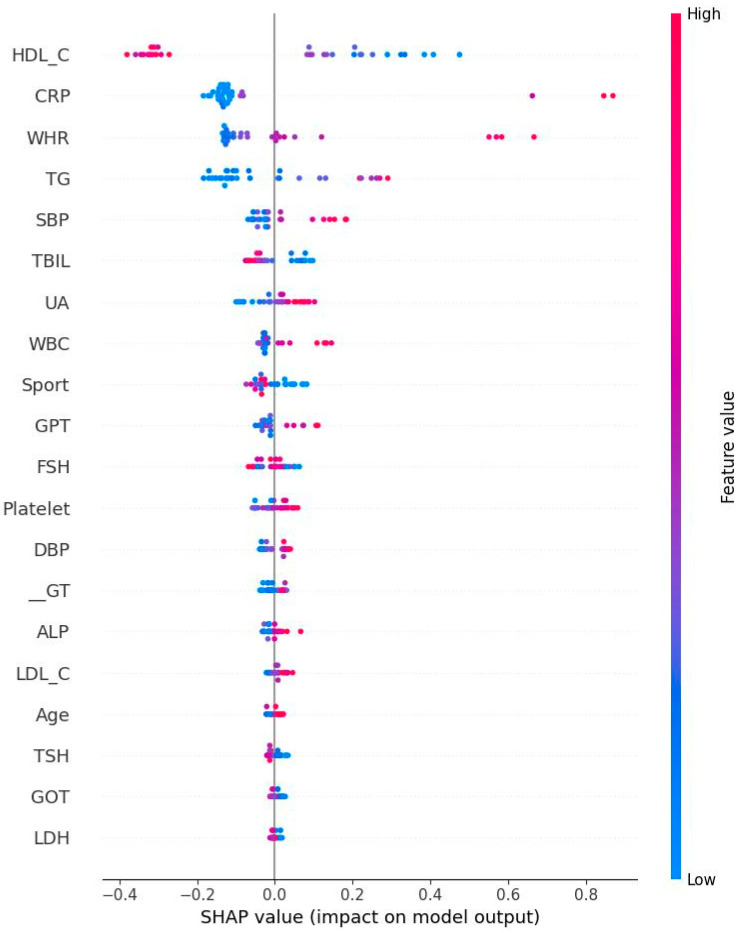
Bee swarm plot derived from Shapley addictive explanation for normal menstrual women. The top features are the most important ones. Red color indicates high impact on the HOMA-IR. HDL-C: High-density lipoprotein cholesterol; CRP: C reactive protein; WHR: Waist–hip ratio; TG: Triglyceride; SBP: Systolic blood pressure; TBIL: Total bilirubin; UA: Uric acid; WBC: Leukocyte; GPT: Serum glutamic pyruvic transaminase; FSH: Follicle-stimulating hormone; DBP: Diastolic blood pressure; r-GT: γ-Glutamyl transpeptidase; ALP: Alkaline phosphatase; LDL-C: Low-density lipoprotein cholesterol; TSH: Thyroid-stimulating hormone; GOT: Serum glutamic oxaloacetic transaminase; LDH: Lactate dehydrogenase.

**Figure 6:**
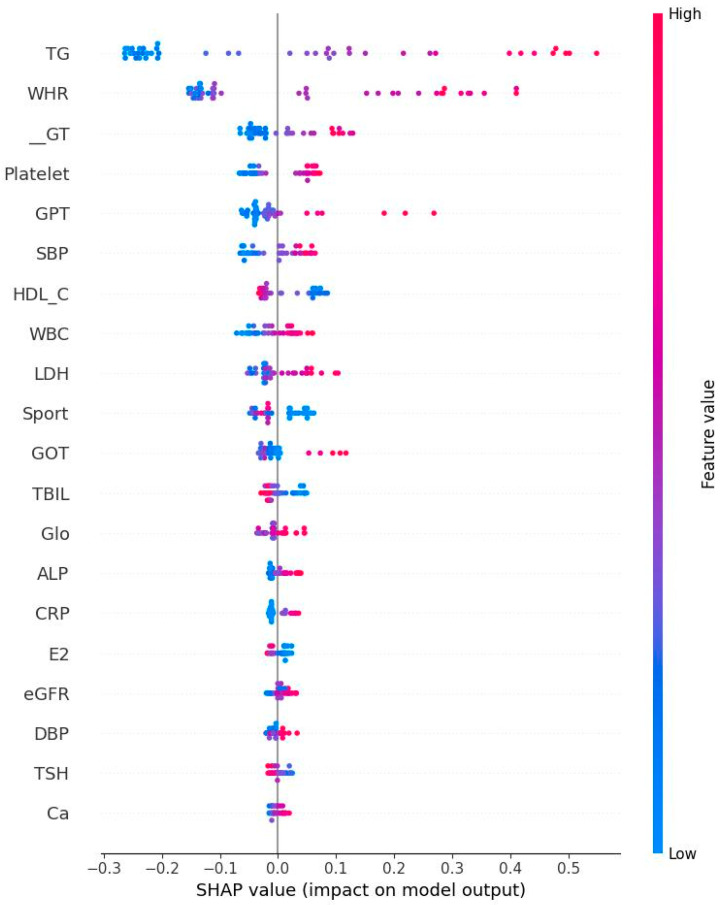
Bee swarm plot derived from Shapley addictive explanation for early menopausal women. The top features are the most important ones. Red color indicates high impact on the HOMA-IR. TG: Triglyceride; WHR: Waist–hip ratio; r-GT: γ-Glutamyl transpeptidase; GPT: Serum glutamic pyruvic transaminase; SBP: Systolic blood pressure; HDL-C: High-density lipoprotein cholesterol; WBC: Leukocyte; LDH: Lactate dehydrogenase; GOT: Serum glutamic oxaloacetic transaminase; TBIL: Total bilirubin; Glo: Globulin; ALP: Alkaline phosphatase; CRP: C reactive protein; E2: Estradiol; eGFR: estimated Glomerular filtration rate; DBP: Diastolic blood pressure; TSH: Thyroid-stimulating hormone; Ca: Plasma calcium concentration.

**Figure 7:**
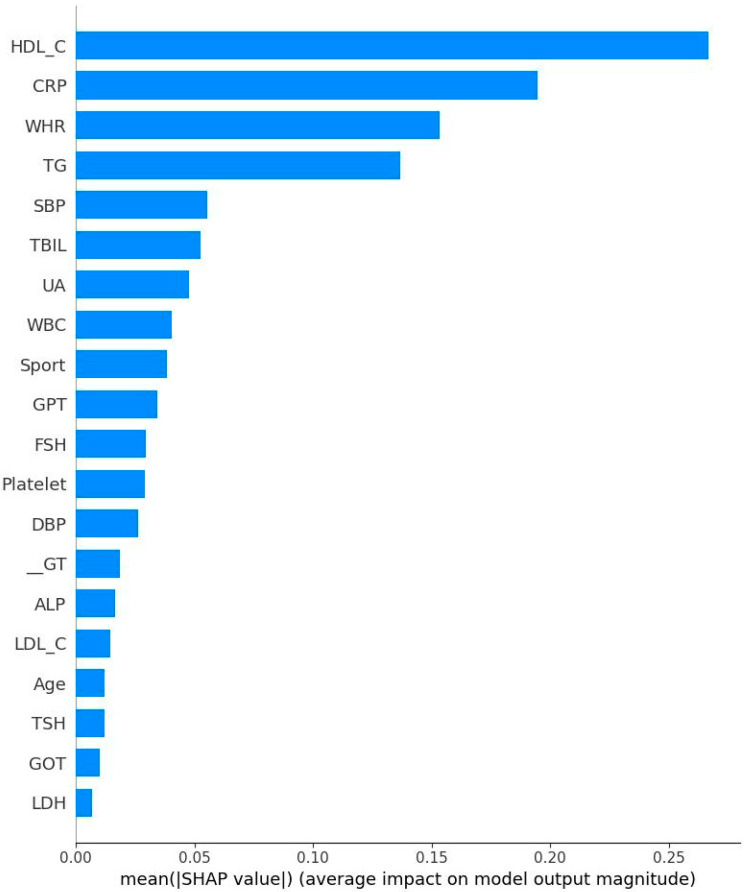
Absolute Shapley addictive values for normal menstrual women. Note: HDL-C: High-density lipoprotein cholesterol; CRP: C reactive protein; WHR: Waist–hip ratio; TG: Triglyceride; SBP: Systolic blood pressure; TBIL: Total bilirubin; UA: Uric acid; WBC: Leukocyte; GPT: Serum glutamic pyruvic transaminase; FSH: Follicle-stimulating hormone; DBP: Diastolic blood pressure; r-GT: γ-Glutamyl transpeptidase; ALP: Alkaline phosphatase; LDL-C: Low-density lipoprotein cholesterol; TSH: Thyroid-stimulating hormone; GOT: Serum glutamic oxaloacetic transaminase; LDH: Lactate dehydrogenase.

**Figure 8:**
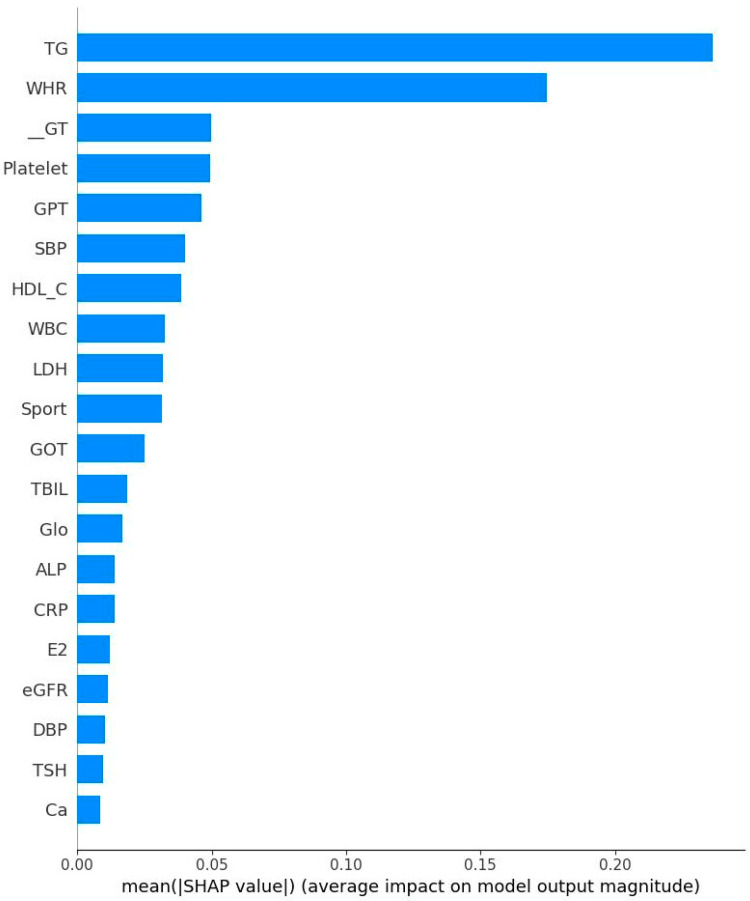
Absolute Shapley addictive values for early menopausal women. Note: TG: Triglyceride; WHR: Waist–hip ratio; r-GT: γ-Glutamyl transpeptidase; GPT: Serum glutamic pyruvic transaminase; SBP: Systolic blood pressure; HDL-C: High-density lipoprotein cholesterol; WBC: Leukocyte; LDH: Lactate dehydrogenase; GOT: Serum glutamic oxaloacetic transaminase; TBIL: Total bilirubin; Glo: Globulin; ALP: Alkaline phosphatase; CRP: C reactive protein; E2: Estradiol; eGFR: estimated Glomerular filtration rate; DBP: Diastolic blood pressure; TSH: Thyroid-stimulating hormone; Ca: Plasma calcium concentration.

Figure 5 and Figure 6 respectively show the Bee swarm of SHAP in NM and EM. For the NM group, the plot shows that HDL-C was the most important factor and the red dot distribution leans to the left, indicating that HDL-C is negatively correlated with IR. For the EM group, TG has the highest impact which drives to a negative relationship for IR. Figure 7 and Figure 8 show the absolute values for Figure 5 and Figure 6, respectively.

## 4. Discussion

This study is the first to use ML to explore risk factors related to IR in NM and EM women. Two factors, i.e., WHC and HDL-C, are found to be significant for both groups. Given the non-longitudinal nature of this study, causal relationships could not be determined, but our findings still provide useful information about the pathophysiology for IR in EM, and can be used to improve early clinical detection of IR.

In line with previous work, the present study only discusses the six most impactful variables for the following reasons: 1. Including too many variables in the main findings can reduce clarity and make it difficult to draw practical clinical conclusions. 2. In all ML models, we observed that the importance scores dropped significantly beyond the top six variables. 3. These top six features were consistently identified (or ranked highly) across multiple algorithms and within both the EM and NM subgroups. 4. A model or interpretation based on the top six variables is easier to translate into clinical tools or decision support systems in real-world primary care settings.

The percentage of importance varies among the four ML methods. Our goal is to identify and compare the most influential features contributing to IR and each model’s built-in method was used to estimate feature importance. For tree-based models (XGBoost and RF), importance was calculated based on the average gain or Gini impurity reduction from splits involving each feature. For regularized regression models (EN), we used the magnitude of standardized model coefficients after regularization, where non-zero coefficients indicated selected features. To enable direct comparison across models with different scales, we normalized all feature importance values by setting the top feature within each model to 100 and scaling the remaining features proportionally. As such, in the XGBoost model, WHR received a normalized importance score of 100, indicating that it was the most predictive feature, not the only one utilized. Other features still contributed to model performance, albeit to a lesser extent. We also reported the average importance across the four ML models to provide a more robust, model-agnostic feature ranking. This approach ensures that the final interpretation reflects consensus patterns across algorithms and avoids overreliance on any single method.

As previously noted, WHR and HDL-C were identified as significant impact factors for both groups, and many previous studies have reported the importance of WHR in the development of IR. For example, Benites-Zapata et al. reported that WHR was positively correlated with HOMA-IR (r = 0307, *p* < 0.001). This relationship is caused by the accumulation of visceral fat which could release inflammatory cytokines and produce oxidative stress, thus increasing the risk of IR [44,45]. Similarly low HDL-C has been repeatedly found to be related to IR. In a review, Siebel et al. pointed out that either acute or chronic increased HDL-C reduced blood glucose levels in T2D by inhibiting cholesteryl ester transfer protein. Higher HDL-C could improve glucose control through many mechanisms such as stimulating insulin secretion or increasing insulin-dependent glucose uptake, leading to decreased risk of IR [46]. The results of the present study further confirm the importance of these two factors.

Aside from WHT and HDL-C, the key impact factors among EM women (from most to least important) are TG, GPT, GOT and LDH.

1. High TG is a well-known hallmark of dyslipidemia and IR [47]. IR is associated with overproduction of very low-density lipoproteins which contain significant amounts of TG [47]. Due to decreased estrogen levels in menopausal women, the liver produces more TG, which could explain the results of the present study [48].
2. GPT could be regarded as a biomarker for IR [49] because that higher GPT levels indicate liver damage which could cause impaired insulin function and glucose metabolism [50]. Menopause is frequently associated with higher GOT and GPT levels due to decreased estrogen levels [51]. Estrogen protects liver cells and helps maintain mitochondrial function. Our finding is consistent with previous results.
3. GOT interacts with IR similarly to GPT. Our result showed an independent relationship to IR. This is not surprising since that GPT is mainly found in liver, kidney, heart, and muscle. GOT is distributed more widely in the body than GPT, including in the brain and blood cells [52], potentially explaining this independent relationship.
4. Subjects with IR have elevated rates of glycolysis, leading to higher production of pyruvate, a precursor for LDH [53]. Estrogen stimulates production of LDH in some tissues and post-menopausal women typically experience a corresponding decrease in LDH levels [54]. Our finding further confirms previous reports related to this area.

In NM women, the other key impact factors (from most to least important) are CRP, TBIL, DBP and WBC.

1. Both CRP and WBC are markers for inflammation and many studies have confirmed the relationship between inflammation and IR [55,56,57]. For example, CRP is picked out to be one of the important features. This does not mean that their relationship is positive. On the contrary, both CRP and HOMA-IR might be correlated negatively. Therefore, the findings in the present study are not surprising. Inflammatory markers are produced in the adipose tissues and liver, inhibiting insulin signaling pathways and leading to IR [58]. Our findings thus are consistent with previous work.
2. Previous studies have established the relationship between blood pressure and IR. Using an euglycemic insulin clamp to quantify IR, Ferrannini et al. reported that DBP is positively correlated with IR (r = 0.18, *p* < 0.05). As for the underlying mechanism, IR could induce the activity of sympathetic nerve and elevate vasoconstrictors such as angiotensin II, leading to increased blood pressure [59]. Again, our conclusion is consistent with past results.
3. TBIL: Pre-menopausal women generally have higher estrogen levels, providing a protective effect for the liver and cardiovascular system. This hormonal environment may help maintain higher or more stable bilirubin levels. After menopause, estrogen levels drop significantly. Previous studies have shown that postmenopausal women often have lower total bilirubin levels compared to premenopausal women. This decrease may be linked to increased risk of hypertension and cardiovascular disease, as bilirubin has antioxidant and anti-inflammatory properties [60].

Reviewing the different impact factors reveals the critical difference between the EM and NM groups is the protective role of estrogen in NM women. Lack of this protection exposes EM women to additional impact factors that are more harmful to the less important factors such as TG, liver enzymes, and LDH. On the other hand, among women with estrogen protection, the major risk factors are the ‘chronic inflammation’. Therefore, pre-menopausal women should focus on maintaining healthy body weight.

Finally, this study assesses the impact of features such as sleeping time, sport area, smoking area, education level, and smoking area. Previous studies have established correlations between some of these features and IR, such as smoking and income [61,62,63]. However, ML approaches are similar to multiple regressions in that the models produced adjust for all confounding features, suggesting the relatively low importance of these features.

Several clinical implications could be derived from the present study.

- Early risk stratifications: WHR and HDL-C could serve as early warning signs for IR.
- Cost-effective screening tools: Variables such was WHR, HDL-C, and CRP could be integrated as a simple composite risk score for integration into electronic health records to flag high-risk individuals for further metabolic assessment.
- Targeted lifestyle interventions: such as exercise, enough sleeping time, and quit smoking.
- As WHR is the strongest predictor, interventions focused on reducing abdominal obesity (via diet, exercise, and behavioral counseling) should be prioritized for women under 50.
- Monitoring inflammatory status in EM Women: EM women, these lose protection of estrogen, so incorporating inflammatory marker monitoring may help guide early preventive strategies during or shortly after the onset of menopause.
- Clinical decision support systems: These findings could inform the development of machine learning-based decision support tools for use in primary care.

Our study is subject to certain limitations. First, this is a cross-sectional study, and determining causal relationships would require a longitudinal study. The cross-sectional nature of this study also raises issues for potential impacts of confounding variables. We also did not perform stratified analysis or sensitivity checks. In regard to the first issue, we applied EN and RF to reduce the impact of collinear or less informative variables. For the second issue, although this study did not primarily focus on stratified analyses or formal sensitivity checks (e.g., stratification by BMI or age groups), we recognize the importance of these methods in future work which will explore stratified modeling (e.g., by BMI or menopausal status) to assess whether associations between predictors and IR differ across subgroups. We will also incorporate propensity score matching or inverse probability weighting to further reduce confounding bias. Finally, while our study presents novel and data-driven insights into IR risk among EM and NM women, further validation is needed to confirm the generalizability of our findings. Our current study used a relatively large, well-characterized cohort (n = 948), and employed multiple, diverse ML algorithms to enhance the robustness of variable selection. Nevertheless, we agree that external validation using independent datasets, longitudinal follow-up to establish temporal relationships, and possibly experimental or interventional studies would provide stronger causal inference and enhance the clinical utility of our model. Future work will aim to replicate these results in larger and multi-centered cohorts and to explore biological mechanisms through which key features—such as WHR, HDL-C, and liver enzymes—interact with estrogen deficiency to influence IR risk. These next steps will help translate our ML-driven findings into clinically actionable strategies. Finally, the present study used exclusively Taiwanese subjects and generalization to other ethnic groups should be performed with caution.

## 5. Conclusions

Using ML, we found that WHR and HDL-C are the common denominators in both EM and NM women, with additional correlations with TG, liver enzymes and LDH for EM women. These results clearly indicate the importance of estrogen protection, suppressing less important factors (TG, liver enzyme, and LDH), and only the stronger inflammatory markers become important (CRP, TBIL, and WBC). Once estrogen’s protection disappears, the suppression of CRP, TBIL, and WBC would become weaker. Since these 3 features are significantly correlated with body weight, for women under 50, reducing body weight is the most important factor in preventing hyperglycemia.

## Figures and Tables

**Table 1:** HOMA-IR, associated independent variables, and their units.

Variables	Unit and Description
Age	Years
Marital status, MS	(1) Unmarried (2) Married
Income level, IL	NTD/year (1) Below USD 200,000 (2) USD 200,001–USD 400,000 (3) USD 400,001–USD 800,000 (4) USD 800,001–USD 1,200,000 (5) USD 1,200,001–USD 1,600,000 (6) USD 1,600,001–USD 2,000,000 (7) More than USD 2,000,000
Education level, Edu.	(1) Illiterate (2) Elementary school (3) Junior high school (4) High school (vocational) (5) Junior college (6) University (7) Graduate school or above
Waist–hip ratio, WHR	Waist circumference/hip circumference
Systolic blood pressure, SBP	mmHg
Diastolic blood pressure, DBP	mmHg
Leukocyte, WBC	×10^3^/μL
Hemoglobin, Hb	×10^6^/μL
Platelets, Plt	×10^3^/μL
Fasting plasma glucose, FPG	mg/dL
Fasting plasma insulin, FPI	uU/mL
Total bilirubin, TBIL	mg/dL
Albumin, Alb	mg/dL
Globulin, Glo	g/dL
Alkaline Phosphatase, ALP	IU/L
Serum glutamic oxaloacetic transaminase (SGOT)	IU/L
Serum glutamic pyruvic transaminase, SGPT	IU/L
Serum γ-glutamyl transpeptidase, γ-GT	IU/L
Lactate dehydrogenase, LDH	mg/dL
estimated Glomerular filtration rate, eGFR	mg/dL
Uric acid, UA	mg/dL
Triglycerides, TG	mg/dL
High-density lipoprotein cholesterol, HDL-C	mg/dL
Low-density lipoprotein cholesterol, LDL-C	mg/dL
Plasma calcium concentration, Ca	mg/dL
Plasma phosphate concentration, P	mg/dL
Thyroid-stimulating hormone, TSH	μIU/mL
C reactive protein, CRP	mg/dL
Follicle-stimulating hormone, FSH	mIU/mL
Estradiol, E2	pg/mL
Drinking area	-
Smoking area	-
Sport area	-
Sleeping hours, SH	(1) 0~4 h (2) 4~6 h (3) 6~7 h (4) 7~8 h (5) 8~9 h (6) more than 9 h
HOMA-IR	FPI (μU/mL) × FPG (mg/dL)/405

**Table 2:** Equations for performance metrics.

Metrics	Description	Calculation Formula
MAPE	Mean Absolute Percentage Error	$MAPE=\frac{1}{n}\sum_{i=1}^{n}\left|\frac{y_{i}-{\hat{y}}_{i}}{y_{i}}\right|\times 100$
SMAPE	Symmetric Mean Absolute Percentage Error	$SMAPE=\frac{1}{n}\sum_{i=1}^{n}\frac{\left|y_{i}-{\hat{y}}_{i}\right|}{\left(\left|y_{i}\right|+\left|{\hat{y}}_{i}\right|\right)/2}\times 100$
RAE	Relative Absolute Error	$RAE=\sqrt{\frac{\sum_{i=1}^{n}{\left(y_{i}-{\hat{y}}_{i}\right)}^{2}}{\sum_{i=1}^{n}{\left(y_{i}\right)}^{2}}}$
RRSE	Root Relative Squared Error	$RRSE=\sqrt{\frac{\sum_{i=1}^{n}{\left(y_{i}-{\hat{y}}_{i}\right)}^{2}}{\sum_{i=1}^{n}{\left(y_{i}-{\hat{y}}_{i}\right)}^{2}}}$
RMSE	Root Mean Squared Error	$RMSE=\sqrt{\frac{1}{n}\sum_{i=1}^{n}{\left(y_{i}-{\hat{y}}_{i}\right)}^{2}}$

**Table 3:** Statistical Comparison of NM and EM Groups via T-Test.

Numeric Variable	Non-Menopause n = 410 Mean ± SD	Early Menopause n = 538 Mean ± SD
Age	47.27 ± 0.19	46.77 ± 0.19
Waist–hip ratio, WHR	0.77 ± 0.00	0.77 ± 0.00
Systolic blood pressure, SBP	111.75 ± 0.82	112.43 ± 0.66
Diastolic blood pressure, DBP	72.28 ± 0.54	72.08 ± 0.47
Leukocyte, WBC	5.52 ± 0.08	5.83 ± 0.06 **
Hemoglobin, Hb	13.56 ± 0.04	12.85 ± 0.06 ***
Platelets, Plt	237.79 ± 2.52	257.33 ± 2.76 ***
Fasting plasma glucose, FPG	101.08 ± 0.85	99.99 ± 0.67
Fasting plasma insulin	7.18 ± 4.33	7.15 ± 4.04
Total bilirubin, TBIL	0.96 ± 0.01	0.90 ± 0.01 **
Albumin, Alb	4.40 ± 0.00	4.32 ± 0.00 ***
Globulin, Glo	3.13 ± 0.01	3.13 ± 0.01
Alkaline phosphatase, ALP	62.44 ± 0.91	52.90 ± 0.70 ***
Serum glutamic oxaloacetic transaminase, SGOT	23.00 ± 0.44	21.67 ± 0.49
Serum glutamic pyruvic transaminase, SGPT	24.32 ± 0.74	22.50 ± 0.77
Serum γ-glutamyl transpeptidase, γ-GT	26.34 ± 1.37	22.79 ± 1.04 *
Lactate dehydrogenase, LDH	165.18 ± 1.61	156.21 ± 1.27 ***
estimated Glomerular filtration rate, eGFR	82.03 ± 0.63	82.79 ± 0.47
Uric acid, UA	4.94 ± 0.05	4.76 ± 0.04 *
Triglycerides, TG	104.79 ± 3.15	92.91 ± 2.28 **
High-density lipoprotein cholesterol, HDL-C	65.29 ± 0.81	63.40 ± 0.64
Low-density lipoprotein cholesterol, LDL-C	121.45 ± 1.63	115.86 ± 1.35 **
Plasma calcium concentration, Ca	9.58 ± 0.01	9.37 ± 0.01 ***
Plasma phosphate concentration, P	4.02 ± 0.02	3.72 ± 0.02 ***
Thyroid-stimulating hormone, TSH	1.77 ± 0.08	1.76 ± 0.04
C reactive protein, CRP	0.20 ± 0.01	0.21 ± 0.01
Follicle-stimulating hormone, FSH	48.39 ± 1.72	17.42 ± 0.99 ***
Estradiol, E2	40.92 ± 4.28	96.30 ± 4.62 ***
HOMA-IR	1.84 ± 0.06	1.81 ± 0.05
Drinking area	1.36 ± 0.33	1.34 ± 0.33
Smoking area	1.29 ± 0.34	0.87 ± 0.26
Sport area	4.52 ± 0.35	4.06 ± 0.24
Range of HOMA-IR	0.246–8.71	0.332–13.68
Ordinal variables	N (%)	N (%)
Marital status (MS)		
(1) Unmarried	90 (24.46)	140 (28.34)
(2) Married	278 (75.54)	354 (71.66)
Categorical variables	N (%)	N (%)
Income level (IL)		
(1) Below USD 200,000	24 (11.37)	31 (10.47)
(2) USD 200,001–USD 400,000	49 (23.22)	64 (21.62)
(3) USD 400,001–USD 800,000	65 (30.81)	91 (30.74)
(4) USD 800,001–USD 1,200,000	44 (20.85)	69 (23.31)
(5) USD 1,200,001–USD 1,600,000	17 (8.06)	20 (6.76)
(6) USD 1,600,001–USD 2,000,000	4 (1.90)	8 (2.70)
(7) More than USD 2,000,000	8 (3.79)	13 (4.39)
Education level (Edu.)		
(1) Illiterate	0 (0.00)	0 (0.00)
(2) Elementary school	3 (0.82)	3 (0.60)
(3) Junior high school	12 (3.28)	12 (2.40)
(4) High school (vocational)	124 (33.88)	124 (24.85)
(5) Junior college	93 (25.41)	140 (28.06)
(6) University	99 (27.05)	165 (33.07)
(7) Graduate school or above	35 (9.56)	55 (11.02)
Sleeping hours (SH)		
(1) 0~4 h	8 (2.01)	8 (1.54)
(2) 4~6 h	120 (30.15)	142 (27.26)
(3) 6~7 h	186 (46.73)	252 (48.37)
(4) 7~8 h	67 (16.83)	100 (19.19)
(5) 8~9 h	16 (4.02)	18 (3.45)
(6) more than 9 h	1 (0.25)	1 (0.19)

Significant difference are shown as the mean; *: *p* < 0.05; **: *p* < 0.01; ***: *p* < 0.001.

**Table 4:** Simple correlation between all continuous independent variables and HOMA-IR.

Menopause						
	age	WHR	SBP	DBP	WBC	Hb
HOMA-IR	−0.07	0.43 ***	0.26 ***	0.24 ***	0.32 ***	0.12 **
	Plt	FPG	TBIL	Alb	Glo	ALP
HOMA-IR	0.25 ***	0.50 ***	−0.24 ***	0.16 ***	0.18 ***	0.25 ***
	SGOT	SGPT	r-GT	LDH	eGFR	UA
HOMA-IR	0.30 ***	0.41 ***	0.31 ***	0.14 ***	0.00	0.34 ***
	TG	HDL-C	LDL-C	Ca	P	TSH
HOMA-IR	0.45 ***	−0.37 ***	0.26 ***	0.11 *	−0.08	0.08
	CRP	FSH	E2	Drinking area	Smoking area	Sport area
HOMA-IR	0.19 ***	−0.09 *	−0.06	−0.05	−0.06	−0.23 ***
Early menopause						
	age	WHR	SBP	DBP	WBC	Hb
HOMA-IR	−0.00	0.50 ***	0.28 ***	0.28 ***	0.33 ***	0.12 **
	Plt	FPG	TBIL	Alb	Glo	ALP
HOMA-IR	0.19 ***	0.50 ***	−0.25 ***	0.14 **	0.10 *	0.16 ***
	SGOT	SGPT	r-GT	LDH	eGFR	UA
HOMA-IR	0.12 *	0.36 ***	0.16 ***	0.02	0.01	0.31 ***
	TG	HDL-C	LDL-C	Ca	P	TSH
HOMA-IR	0.37 ***	−0.37 ***	0.20 ***	0.04	−0.09	0.04
	CRP	FSH	E2	Drinking area	Smoking area	Sport area
HOMA-IR	0.28 ***	−0.15 **	−0.04	−0.04	−0.01	−0.03

Note: Data are shown as the mean; HOMA-IR: Homeostatic Model Assessment for Insulin Resistance; WHR: Waist–hip ratio; SBP: Systolic blood pressure; DBP: Diastolic blood pressure; WBC: Leukocyte; Hb: Hemoglobin; Plt: Platelets; FPG: Fasting plasma glucose, TBIL: Total bilirubin; Alb: Albumin; Glo: Globulin; ALP: Alkaline phosphatase; SGOT: Serum glutamic oxaloacetic transaminase; SGPT: Serum glutamic pyruvic transaminase; r-GT: γ-Glutamyl transpeptidase; LDH: Lactate dehydrogenase; eGFR: estimated Glomerular filtration rate; UA: Uric acid; TG: Triglyceride; HDL-C: High-density lipoprotein cholesterol; LDL-C: Low-density lipoprotein cholesterol; Ca: Plasma calcium concentration, P: Plasma phosphate concentration; TSH: Thyroid-stimulating hormone; CRP: C reactive protein; FSH: Follicle-stimulating hormone; E2: Estradiol. Significant difference are shown as the mean; *: *p* < 0.05; **: *p* < 0.01; ***: *p* < 0.001.

**Table 5:** Average performance of the four ML methods and the MLR method.

Menopause					
Methods	MAPE	SMAPE	RAE	RRSE	RMSE
MLR	0.54	0.4293	0.974	0.9549	0.9729
RF	0.4785	0.3567	0.8572	0.8592	0.8753
SGB	0.5029	0.3659	0.8852	0.9107	0.9278
XGBoost	0.5384	0.3636	0.9182	0.927	0.9444
EN	0.4854	0.3671	0.8731	0.8514	0.8673
Early menopause					
Methods	MAPE	SMAPE	RAE	RRSE	RMSE
MLR	0.5063	0.5345	1.063	1.1386	1.1046
RF	0.4196	0.3365	0.7619	0.8308	0.806
SGB	0.457	0.3782	0.9129	0.9724	0.9433
XGBoost	0.4862	0.3981	0.9281	1.0127	0.9824
EN	0.4765	0.3772	0.8438	0.8815	0.8551

Note: Data are shown as the mean; MLR: multiple linear regression; RF: random forest; SGB: stochastic gradient boosting; XGBoost: eXtreme Gradient Boosting; EN: Elastic Net; MAPE: Mean Absolute Percentage Error; SMAPE: symmetric mean absolute percentage error; RAE: relative absolute error; RRSE: root relative squared error; RMSE: root mean squared error.

**Table 6:** Importance percentage derived from four different ML methods and their averages (Normal Menopause).

	RF	SGB	XGBoost	EN	Average
Age	7.59	22.02	9.82	0.00	9.86
MS	1.02	0.00	0.00	0.00	0.26
Income	2.57	0.64	1.96	0.00	1.29
Edu	1.99	6.06	5.87	0.00	3.48
WHR	71.50	50.64	100.00	100.00	80.54
SBP	15.53	22.39	19.47	0.00	14.35
DBP	19.52	1.65	4.63	0.23	6.51
WBC	15.27	20.13	13.81	1.17	12.60
Hb	20.85	10.17	32.12	0.02	15.79
Plt	13.06	1.77	11.44	0.05	6.58
TBIL	9.89	3.38	2.60	0.00	3.97
Alb	3.77	4.23	2.98	0.00	2.75
Glo	16.07	6.96	0.29	0.00	5.83
ALP	6.76	0.74	2.72	0.00	2.56
SGOT	25.03	40.76	46.26	0.00	28.01
SGPT	67.80	29.96	31.45	0.67	32.47
r-GT	10.75	2.95	2.94	0.00	4.16
LDH	16.09	21.11	30.28	0.01	16.87
eGFR	9.84	2.92	2.88	0.00	3.91
UA	7.49	1.96	9.92	0.90	5.07
TG	100.00	100.00	84.22	0.26	71.12
HDL-C	18.15	33.87	40.01	0.20	23.06
LDL-C	22.95	9.97	23.68	0.00	14.15
Ca	10.12	8.29	14.48	10.50	10.85
P	8.91	3.89	3.93	0.00	4.18
TSH	13.38	16.52	11.56	0.00	10.37
CRP	7.44	19.05	7.33	7.81	10.41
FSH	8.20	5.72	8.24	0.00	5.54
E2	7.45	11.95	4.94	0.00	6.09
Drinking area	0.27	0.00	0.00	0.00	0.07
Smoking area	0.00	0.00	0.00	0.00	0.00
Sport area	6.55	8.89	34.67	0.27	12.60
SH	0.96	1.52	0.00	0.00	0.62

Note: The full names of the variables and ML methods abbreviations are shown as the mean; RF: random forest; SGB: stochastic gradient boosting; XGBoost: eXtreme Gradient Boosting; EN: Elastic Net; MS: Marriage status; Edu: Education level; WHR: Waist–hip ratio; SBP: Systolic blood pressure; DBP: Diastolic blood pressure; WBC: Leukocyte; Hb: Hemoglobin; Plt: Platelets; TBIL: Total bilirubin; Alb: Albumin; Glo: Globulin; ALP: Alkaline phosphatase; SGOT: Serum glutamic oxaloacetic transaminase; SGPT: Serum glutamic pyruvic transaminase; r-GT: γ-Glutamyl transpeptidase; LDH: Lactate dehydrogenase; eGFR: estimated Glomerular filtration rate; UA: Uric acid; TG: Triglyceride; HDL-C: High-density lipoprotein cholesterol; LDL-C: Low-density lipoprotein cholesterol; Ca: Plasma calcium concentration, P: Plasma phosphate concentration; TSH: Thyroid-stimulating hormone; CRP: C reactive protein; FSH: Follicle-stimulating hormone; E2: Estradiol; SH: Sleeping hours.

**Table 7:** Importance percentage of importance derived from four different ML methods and their averages (non-menopause).

	RF	SGB	XGBoost	EN	Average
Age	7.43	7.15	16.04	0.00	7.66
MS	0.64	0.00	0.00	0.00	0.16
Income	3.68	0.00	5.13	0.00	2.20
Edu	7.74	0.00	35.49	0.00	10.81
WHR	72.08	73.84	100.00	100.00	86.48
SBP	18.36	0.00	3.80	0.01	5.54
DBP	14.35	42.15	53.20	0.25	27.49
WBC	36.24	32.52	18.28	0.80	21.96
Hb	5.46	0.00	2.78	0.00	2.06
Plt	23.41	3.50	12.71	0.00	9.91
TBIL	37.07	66.32	71.80	10.62	46.45
Alb	6.69	0.00	3.27	0.00	2.49
Glo	3.68	0.00	11.77	0.00	3.86
ALP	8.35	0.00	5.13	0.00	3.37
SGOT	5.51	8.56	11.19	0.00	6.32
SGPT	18.94	12.56	23.40	0.25	13.79
r-GT	7.12	0.00	5.38	0.00	3.13
LDH	7.89	0.00	8.90	0.00	4.20
eGFR	7.24	2.17	2.92	0.00	3.08
UA	11.29	25.50	15.12	0.02	12.98
TG	31.26	41.57	9.50	0.00	20.58
HDL-C	48.48	50.99	91.22	0.16	47.71
LDL-C	10.78	12.59	47.06	0.03	17.62
Ca	5.22	0.00	10.37	0.00	3.90
P	5.09	2.68	4.01	0.00	2.95
TSH	9.96	13.92	25.46	0.00	12.34
CRP	100.00	100.00	83.58	2.41	71.50
FSH	6.98	0.00	3.86	0.00	2.71
E2	3.96	6.60	11.94	0.00	5.63
Drinking area	0.23	0.00	0.00	0.00	0.06
Smoking area	0.00	0.00	0.00	0.00	0.00
Sport area	7.35	0.00	6.74	0.00	3.52
SH	1.56	0.00	0.00	0.00	0.39

Note: The full names of the variables and ML methods abbreviations are shown as the mean; RF: random forest; SGB: stochastic gradient boosting; XGBoost: eXtreme Gradient Boosting; EN: Elastic Net; MS: Marriage status; Edu: Education level; WHR: Waist–hip ratio; SBP: Systolic blood pressure; DBP: Diastolic blood pressure; WBC: Leukocyte; Hb: Hemoglobin; Plt: Platelets; TBIL: Total bilirubin; Alb: Albumin; Glo: Globulin; ALP: Alkaline phosphatase; SGOT: Serum glutamic oxaloacetic transaminase; SGPT: Serum glutamic pyruvic transaminase; r-GT: γ-Glutamyl transpeptidase; LDH: Lactate dehydrogenase; eGFR: estimated Glomerular filtration rate; UA: Uric acid; TG: Triglyceride; HDL-C: High-density lipoprotein cholesterol; LDL-C: Low-density lipoprotein cholesterol; Ca: Plasma calcium concentration, P: Plasma phosphate concentration; TSH: Thyroid-stimulating hormone; CRP: C reactive protein; FSH: Follicle-stimulating hormone; E2: Estradiol; SH: Sleeping hours.

## Data Availability

Data available on request due to privacy/ethical restrictions.

## References

[1] World Health Organization Urgent Action Needed as Global Diabetes Cases Increase Four-Fold Over Past Decades. https://www.who.int/news/item/13-11-2024-urgent-action-needed-as-global-diabetes-cases-increase-four-fold-over-past-decades.

[2] Banday M.Z., Sameer A.S., Nissar S. (2020). Pathophysiology of diabetes: An overview. Avicenna J. Med..

[3] Chow S.N., Huang C.C., Lee Y.T. (1997). Demographic characteristics and medical aspects of menopausal women in Taiwan. J. Formos. Med. Assoc. Taiwan Yi Zhi.

[4] Shen T.-Y., Strong C., Yu T. (2020). Age at menopause and mortality in Taiwan: A cohort analysis. Maturitas.

[5] Al-Maaitah A.M., Al-Nasser M.A., Al-Khateeb M.A. (2012). Premature menopause: A review. Menopause Int..

[6] Merck Manuals Premature menopause. In Merck Manuals. https://www.msdmanuals.com/home/women-s-health-issues/menstrual-disorders-and-abnormal-vaginal-bleeding/premature-menopause.

[7] Healthline Causes of Early Menopause. https://www.healthline.com/health/menopause/causes-early.

[8] Stachowiak G., Pertyński T., Pertyńska-Marczewska M. (2015). Metabolic disorders in menopause. Menopause Rev./Przegląd Menopauzalny.

[9] Hevener A.L., Clegg D.J., Mauvais-Jarvis F. (2015). Impaired estrogen receptor action in the pathogenesis of the metabolic syndrome. Mol. Cell. Endocrinol..

[10] Lin K.C., Tsay S.L., Tsai S.T., Kuo S.C., Chou P. (2007). Interrelationship between insulin resistance and menopause on the metabolic syndrome and its individual component among nondiabetic women in the kinmen study. Am. J. Med. Sci..

[11] Haider A.W., Larson M.G., Franklin S.S., Levy D. (2003). Systolic blood pressure, diastolic blood pressure, and pulse pressure as predictors of risk for congestive heart failure in the Framingham Heart Study. Ann. Intern. Med..

[12] Yazdkhasti M., Jafarabady K., Shafiee A., Omran S.P., Mahmoodi Z., Esmaeilzadeh S., Babaheidari T.B., Kabir K., Peisepar M., Bakhtiyari M. (2024). The association between age of menopause and type 2 diabetes: A systematic review and meta-analysis. Nutr. Metab..

[13] Nichols A.R., Chavarro J.E., Oken E. (2024). Reproductive risk factors across the female lifecourse and later metabolic health. Cell Metab..

[14] Xing Z., Chen H., Alman A.C. (2024). Discriminating insulin resistance in middle-aged nondiabetic women using machine learning approaches. AIMS Public Health.

[15] Metwally A.A., Heydari A.A., McDuff D., Solot A., Esmaeilpour Z., Faranesh A.Z., Zhou M., Savage D.B., Heneghan C., Patel S. (2025). Insulin Resistance Prediction from Wearables and Routine Blood Biomarkers. arXiv.

[16] Gao W., Deng Z., Gong Z., Jiang Z., Ma L. (2025). AI-driven Prediction of Insulin Resistance in Normal Populations. arXiv.

[17] Liu C.-H., Chang C.-F., Chen I.-C., Lin F.-M., Tzou S.-J., Hsieh C.-B., Chu T.-W., Pei D. (2024). Machine learning prediction of prediabetes in a young male Chinese cohort with 5.8-year follow-up. Diagnostics.

[18] MJ Health Resource Center MJ Health Screening Equipment Use and Replacement Records. MJ Health Resource Center. http://www.mjhrf.org/upload/user/files/MJHRF-TR-06%20Screening%20Equipment.pdf.

[19] Tzou S.-J., Peng C.-H., Huang L.-Y., Chen F.-Y., Kuo C.-H., Wu C.-Z., Chu T.-W. (2023). Comparison between linear regression and four different machine learning methods in selecting risk factors for osteoporosis in a Chinese female-aged cohort. J. Chin. Med. Assoc..

[20] Tseng C.J., Lu C.J., Chang C.C., Chen G.D., Cheewakriangkrai C. (2017). Integration of data mining classification techniques and ensemble learning to identify risk factors and diagnose ovarian cancer recurrence. Artif. Intell. Med..

[21] Chang C.C., Chen S.H. (2019). Developing a novel machine learning-based classification scheme for predicting SPCs in breast cancer survivors. Front. Genet..

[22] Shih C.C., Lu C.J., Chen G.D., Chang C.C. (2020). Risk prediction for early chronic kidney disease: Results from an adult health examination program of 19,270 individuals. Int. J. Environ. Res. Public Health.

[23] Lee H.T., Shin J., Min S.Y., Lim Y.H., Kim K.S., Kim S.G., Kim J.H., Kim J.K. (2015). The relationship between bone mineral density and blood pressure in the Korean elderly population: The Korea National Health and Nutrition Examination Survey, 2008–2011. Clin. Exp. Hypertens..

[24] Chang C.C., Yeh J.H., Chen Y.M., Jhou M.J., Lu C.J. (2021). Clinical predictors of prolonged hospital stay in patients with myasthenia gravis: A study using machine learning algorithms. J. Clin. Med..

[25] Chang C.C., Huang T.H., Shueng P.W., Chen S.H., Chen C.C., Lu C.J., Tseng Y.J. (2021). Developing a stacked ensemble-based classification scheme to predict second primary cancers in head and neck cancer survivors. Int. J. Environ. Res. Public Health.

[26] Chiu Y.L., Jhou M.J., Lee T.S., Lu C.J., Chen M.S. (2021). Health data-driven machine learning algorithms applied to risk indicators assessment for chronic kidney disease. Risk Manag. Healthc. Policy.

[27] Wu T.E., Chen H.A., Jhou M.J., Chen Y.N., Chang T.J., Lu C.J. (2020). Evaluating the effect of topical atropine use for myopia control on intraocular pressure by using machine learning. J. Clin. Med..

[28] Wu C.W., Shen H.L., Lu C.J., Chen S.H., Chen H.Y. (2021). Comparison of different machine learning classifiers for glaucoma diagnosis based on Spectralis OCT. Diagnostics.

[29] Wu C.Z., Huang L.Y., Chen F.Y., Kuo C.H., Yeih D.F. (2023). Using machine learning to predict abnormal carotid intima-media thickness in type 2 diabetes. Diagnostics.

[30] Breiman L. (2001). Random forests. Mach. Learn..

[31] Calle M., Urrea V. (2011). Letter to the editor: Stability of random forest importance measures. Brief. Bioinform..

[32] Friedman J.H. (2001). Greedy function approximation: A gradient boosting machine. Ann. Stat..

[33] Friedman J.H. (2002). Stochastic gradient boosting. Comput. Stat. Data Anal..

[34] Chen T., Guestrin C. XGBoost: A scalable tree boosting system. Proceedings of the 22nd ACM SIGKDD International Conference on Knowledge Discovery and Data Mining.

[35] Torlay L., Perrone-Bertolotti M., Thomas E., Baciu M. (2017). Machine learning–XGBoost analysis of language networks to classify patients with epilepsy. Brain Inform..

[36] Tay J.K., Narasimhan B., Hastie T. (2023). Elastic net regularization paths for all generalized linear models. J. Stat. Softw..

[37] R Core Team (2021). R: A Language and Environment for Statistical Computing. R Foundation for Statistical Computing. https://www.R-project.org/.

[38] RStudio Team (2018). RStudio: Integrated Development for R. RStudio, PBC. http://www.rstudio.com/.

[39] Breiman L., Cutler A., Liaw A., Wiener M. (2018). randomForest: Breiman and Cutler’s Random Forests for Classification and Regression (R Package Version 4.6-14). https://CRAN.R-project.org/package=randomForest.

[40] Greenwell B., Boehmke B., Cunningham J. (2020). Gbm: Generalized Boosted Regression Models (R Package Version 2.1.8). https://CRAN.R-project.org/package=gbm.

[41] Therneau T., Atkinson B. (2019). Rpart: Recursive Partitioning and Regression Trees (R Package Version 4.1.15). https://CRAN.R-project.org/package=rpart.

[42] Chen T., He T., Benesty M., Khotilovich V., Tang Y., Cho H., Chen K., Mitchell R., Cano I., Zhou T. (2021). Xgboost: Extreme Gradient Boosting (R Package Version 1.5.0.2). https://CRAN.R-project.org/package=xgboost.

[43] Kuhn M. (2021). Caret: Classification and Regression Training (R Package Version 6.0-90). https://CRAN.R-project.org/package=caret.

[44] Sirbu A.E., Buburuzan L., Kevorkian S., Martin S., Barbu C., Copaescu C., Smeu B., Fica S. (2018). Adiponectin expression in visceral adiposity is an important determinant of insulin resistance in morbid obesity. Endokrynol. Pol..

[45] Frayn K.N. (2000). Visceral fat and insulin resistance: Causative or correlative?. Br. J. Nutr..

[46] Siebel A.L., Heywood S.E., Kingwell B.A. (2015). HDL and glucose metabolism: Current evidence and therapeutic potential. Front. Pharmacol..

[47] Howard B.V. (1999). Insulin resistance and lipid metabolism. Am. J. Cardiol..

[48] Medical News Today Menopause and Cholesterol: What You Need to Know. Medical News Today. https://www.medicalnewstoday.com/articles/menopause-and-cholesterol.

[49] Bonnet F., Ducluzeau P.H., Gastaldelli A., Laville M., Anderwald C.H., Konrad T., Mari A., Balkau B., RISC Study Group (2011). Liver enzymes are associated with hepatic insulin resistance, insulin secretion, and glucagon concentration in healthy men and women. Diabetes.

[50] Kälsch J., Bechmann L., Heider D., Best J., Manka P., Kälsch H., Sowa J.-P., Moebus S., Slomiany U., Jöckel K.-H. (2015). Normal liver enzymes are correlated with the severity of metabolic syndrome in a large population-based cohort. Sci. Rep..

[51] Aziz N.M. (2021). Effect of postmenopausal on some liver enzymes in Kirkuk women. Al-Qadisiyah J. Pure Sci..

[52] Hernández Pérez J.M., Blanco I., Jesús Sánchez Medina A., Díaz Hernández L., Antonio Pérez Pérez J. (2020). Serum levels of glutamate-pyruvate transaminase, glutamate-oxaloacetate transaminase, and gamma-glutamyl transferase in 1494 patients with various genotypes for the alpha-1 antitrypsin gene. J. Clin. Med..

[53] Maschari D., Saxena G., Law T.D., Walsh E., Campbell M.C., Consitt L.A. (2022). Lactate-induced lactylation in skeletal muscle is associated with insulin resistance in humans. Front. Physiol..

[54] Nagai M.A., Sonohara S., Brentani M.M. (1988). Estrogen control of lactate dehydrogenase isoenzyme-5 in human breast cancer. Int. J. Cancer.

[55] Fizelova M., Jauhiainen R., Kangas A.J., Soininen P., Ala-Korpela M., Kuusisto J., Laakso M., Stancáková A. (2017). Differential associations of inflammatory markers with insulin sensitivity and secretion: The prospective METSIM study. J. Clin. Endocrinol. Metab..

[56] Greenfield J.R., Campbell L.V. (2006). Relationship between inflammation, insulin resistance, and type 2 diabetes: ‘Cause or effect’?. Curr. Diabetes Rev..

[57] Mahdiani A., Kheirandish M., Bonakdaran S. (2019). Correlation between white blood cell count and insulin resistance in type 2 diabetes. Curr. Diabetes Rev..

[58] de Luca C., Olefsky J.M. (2008). Inflammation and insulin resistance. FEBS Lett..

[59] Ferrannini E., Natali A., Capaldo B., Lehtovirta M., Jacob S., Yki-Järvinen H. (1997). Insulin resistance, hyperinsulinemia, and blood pressure: Role of age and obesity. European Group for the Study of Insulin Resistance (EGIR). Hypertension.

[60] He Z., Zhang S., Thio C., Wang Y., Li M., Wu Y., Lin R., Liu Z., Snieder H., Zhang Q. (2022). Serum total bilirubin and new-onset hypertension in perimenopausal women: A cross-sectional study. Menopause.

[61] Facchini F.S., Hollenbeck C.B., Jeppesen J., Chen Y.D.I., Reaven G.M. (1992). Insulin resistance and cigarette smoking. Lancet.

[62] Snijder M.B., Dekker J.M., Visser M., Yudkin J.S., Stehouwer C.D., Bouter L.M., Heine R.J., Nijpels G., Seidell J.C. (2003). Larger thigh and hip circumferences are associated with better glucose tolerance: The Hoorn study. Obes. Res..

[63] Stringhini S., Carmeli C., Jokela M., Avendaño M., Muennig P., Guida F., Ricceri F., d’Errico A., Barros H., Bochud M. (2017). Socioeconomic status and the 25× 25 risk factors as determinants of premature mortality: A multicohort study and meta-analysis of 1· 7 million men and women. Lancet.

